# A prospective multicenter study on genome wide associations to ranibizumab treatment outcome for age-related macular degeneration

**DOI:** 10.1038/s41598-017-09632-0

**Published:** 2017-08-23

**Authors:** Kenji Yamashiro, Keisuke Mori, Shigeru Honda, Mariko Kano, Yasuo Yanagi, Akira Obana, Yoichi Sakurada, Taku Sato, Yoshimi Nagai, Taiichi Hikichi, Yasushi Kataoka, Chikako Hara, Yasurou Koyama, Hideki Koizumi, Munemitsu Yoshikawa, Masahiro Miyake, Isao Nakata, Takashi Tsuchihashi, Kuniko Horie-Inoue, Wataru Matsumiya, Masashi Ogasawara, Ryo Obata, Seigo Yoneyama, Hidetaka Matsumoto, Masayuki Ohnaka, Hirokuni Kitamei, Kaori Sayanagi, Sotaro Ooto, Hiroshi Tamura, Akio Oishi, Sho Kabasawa, Kazuhiro Ueyama, Akiko Miki, Naoshi Kondo, Hiroaki Bessho, Masaaki Saito, Hidenori Takahashi, Xue Tan, Keiko Azuma, Wataru Kikushima, Ryo Mukai, Akihiro Ohira, Fumi Gomi, Kazunori Miyata, Kanji Takahashi, Shoji Kishi, Hiroyuki Iijima, Tetsuju Sekiryu, Tomohiro Iida, Takuya Awata, Satoshi Inoue, Ryo Yamada, Fumihiko Matsuda, Akitaka Tsujikawa, Akira Negi, Shin Yoneya, Takeshi Iwata, Nagahisa Yoshimura

**Affiliations:** 1 Department of Ophthalmology and Visual Sciences, Kyoto University Graduate School of Medicine, Sakyo Kyoto, 606-8507 Japan; 2 Department of Ophthalmology, Otsu Red Cross Hospital, Otsu Shiga, 520-8511 Japan; 3 Department of Ophthalmology, Saitama Medical University, Iruma Saitama, 350-0495 Japan; 4 Department of Ophthalmology, International University of Health and Welfare, Nasu-Shiobara Tochigi, 329-2763 Japan; 5 Department of Surgery, Division of Ophthalmology, Kobe University Graduate School of Medicine, Chuo Kobe, 650-0017 Japan; 6 Department of Ophthalmology, Fukushima Medical University School of Medicine, Fukushima, 960-1247 Japan; 7 Department of Ophthalmology, Tokyo Women’s Medical University, Yachiyo Medical Center, Chiba, 276-0046 Japan; 8 Department of Ophthalmology, Graduate School of Medicine and Faculty of Medicine, The University of Tokyo, Tokyo, 113-0033 Japan; 9 Ophthalmology and Visual Sciences Program, Duke-NUS Medical School, National University of Singapore, Singapore, 119077 Singapore; 10 Singapore National Eye Centre, Singapore Eye Research Institute, Singapore, 168751 Singapore; 11 Department of Ophthalmology, Seirei Hamamatsu General Hospital, Shizuoka, 430-8558 Japan; 12 Department of Ophthalmology, Faculty of Medicine, University of Yamanashi, Chuo Yamanashi, 409-3898 Japan; 13 Department of Ophthalmology, Gunma University School of Medicine, Gunma, 371-0034 Japan; 14 Takasaki Sato Eye Clinic, Gunma, 370-0036 Japan; 15 Department of Ophthalmology, Kansai Medical University, Osaka, 573-1191 Japan; 16 Ohtsuka Eye Hospital, Sapporo, 001-0016 Japan; 17 Hikichi Eye Clinic, Sapporo, 060-0807 Japan; 18 Miyata Ophthalmic Hospital, Miyazaki, 885-0051 Japan; 19 Department of Ophthalmology, Osaka University Graduate School of Medicine, Osaka, 565-0871 Japan; 20 Department of Ophthalmology, Shimane University Faculty of Medicine, Shimane, 693-0021 Japan; 21 Department of Ophthalmology, Tokyo Women’s Medical University, School of Medicine, Tokyo, 162-8666 Japan; 22 Division of Gene Regulation and Signal Transduction, Research Center for Genomic Medicine, Saitama Medical University, Hidaka Saitama, 350-1241 Japan; 23 Department of Ophthalmology and Visual Sciences, Akita University Graduate School of Medicine, Akita, 010-8543 Japan; 24 Department of Ophthalmology, Jichi Medical University, Tochigi, 329-0498 Japan; 25 Department of Ophthalmology, Hyogo College of Medicine, Hyogo, 663-8501 Japan; 26 Maebashi Central Eye Clinic, Gunma, 371-0031 Japan; 27 Department of Diabetes, Endocrinology and Metabolism, International University of Health and Welfare Hospital, 537-3 Iguchi, Nasu-Shiobara Tochigi, 329-2763 Japan; 28 Center for Genomic Medicine, Kyoto University Graduate School of Medicine, Kyoto, 606-8507 Japan; 29 Department of Ophthalmology, Kagawa University Faculty of Medicine, Miki, Kagawa 761-0793 Japan; 30 Division of Molecular and Cellular Biology, National Institute of Sensory Organs, National Hospital Organization Tokyo Medical Center, Tokyo, 152-8902 Japan

## Abstract

We conducted a genome-wide association study (GWAS) on the outcome of anti-VEGF treatment for exudative age-related macular degeneration (AMD) in a prospective cohort. Four hundred and sixty-one treatment-naïve AMD patients were recruited at 13 clinical centers and all patients were treated with 3 monthly injections of ranibizumab followed by pro re nata regimen treatment for one year. Genomic DNA was collected from all patients for a 2-stage GWAS on achieving dry macula after the initial treatment, the requirement for an additional treatment, and visual acuity changes during the 12-month observation period. In addition, we evaluated 9 single-nucleotide polymorphisms (SNPs) in 8 previously reported AMD-related genes for their associations with treatment outcome. The discovery stage with 256 patients evaluated 8,480,849 SNPs, but no SNPs showed genome-wide level significance in association with treatment outcomes. Although SNPs with P-values of <5 × 10^−6^ were evaluated in replication samples of 205 patients, no SNP was significantly associated with treatment outcomes. Among AMD-susceptibility genes, rs10490924 in *ARMS2/HTRA1* was significantly associated with additional treatment requirement in the discovery stage (*P* = 0.0023), and pooled analysis with the replication stage further confirmed this association (*P* = 0.0013). *ARMS2/HTRA1* polymorphism might be able to predict the frequency of injection after initial ranibizumab treatment.

## Introduction

Age-related macular degeneration (AMD) is one of the most common causes of severe visual impairment in the world. Anti-vascular endothelial growth factor (VEGF) treatment has considerably improved treatment outcomes for exudative AMD. However, long-term treatment outcomes are not always favorable^1–3^. Although continuous monthly injection of anti-VEGF treatment can improve visual acuity and maintain the improved visual acuity in most cases, it can cause retinal pigment epithelium (RPE) atrophy and visual loss^4–6^. Pro ne nata (PRN) regimen aims to follow-up cases without treatment until recurrence of the exudative change after successful initial treatment and perform additional treatment for the recurrence. Strict monthly follow-up and prompt additional treatment for recurrence are indispensable for maintaining improved visual acuity and delaying additional treatment can cause damage to retinal cells and visual loss. To prevent the delay of additional treatment and to pre-empt the laborious procedure of monthly follow-up for both patients and physicians, proactive treatment regimens, such as the treat-and-extend (TAE) regimen, has become a widely used strategy. In proactive treatment regimens, continuous treatment is given to eyes with dried maculae after initial treatments. Although the effects of anti-VEGF drugs on dry maculae have not been thoroughly evaluated, redundant treatment might cause RPE atrophy and visual loss^4–6^. Because one-third to one-fourth of patients with AMD were reported to not require additional treatment after initial treatment to maintain dry maculae over one year^7, 8^, a proactive treatment regimen should be avoided for such patients.

Genetic information would be useful for choosing the best treatment strategy for individual patients. Many studies have been conducted to investigate genetic associations of treatment response with anti-VEGF treatment or photodynamic therapy for AMD. Several studies with candidate gene approaches have demonstrated positive associations of AMD-related genes with treatment outcome^9–13^. However, more studies have reported no associations between AMD-related genes and treatment outcomes^14–24^. So far, genome-wide association study (GWAS) has rarely been conducted to find genes associated with treatment responses for AMD, although GWASs might be useful for identifying non-AMD-related genes associated with treatment responses, which could help determine the best treatment strategy for individual patients to enable personalized medicine.

The odds ratio of a genetic polymorphism must be much higher or much lower than 1.0 to be useful in predicting an effective treatment strategy. In addition, when single-nucleotide polymorphisms (SNPs) are used for personalized medicine, SNPs with relatively high minor allele frequencies are useful because more patients with the minor allele would be predicted to have the different treatment outcome. We conducted the ANGEL (ANalysis of GEnotypes associated with the treatment effects of Lucentis for age-related macular degeneration) study in Japan to perform a GWAS on the outcome of anti-VEGF treatment in a prospective cohort.

## Results

Between September 2011 and December 2014, we enrolled 461 patients at 13 clinical centers in Japan. We tested 256 samples from patients enrolled in this study between September 2011 and October 2012 for the discovery stage, and the remaining 205 patient samples for the replication stage. The fixed dataset for the discovery stage consisted of 8,480,849 SNPs from 255 individuals. The discovery samples consisted of 178 male patients and 78 female patients, while the replication samples consisted of 135 male patients and 70 female patients (*P* = 0.42). The average age was 73.6 ± 7.9 years old in the discovery stage and 74.5 ± 7.7 years old in the replication stage (*P* = 0.11). Four participants did not complete the loading treatment and 8 participants dropped out of the study during the PRN period among the discovery samples, and 1 participant did not complete the loading treatment and 13 participants dropped out from the study during the PRN period among the replication samples.

**Figure 1:**
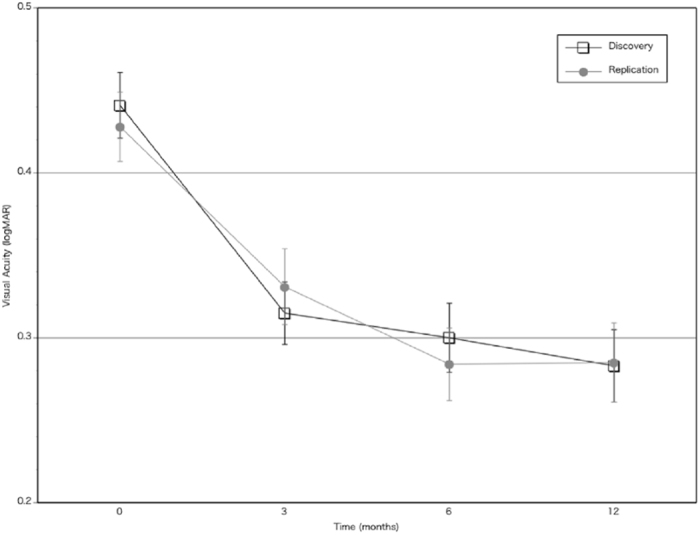
Visual acuity changes during 12 months of ranibizumab treatment in the discovery group (N = 256) and replication group (N = 205). Error bars indicate standard errors of the means.

Visual acuity changes of AMD eyes treated with ranibizumab are shown in Fig. 1. The discovery group and replication group showed similar visual acuity changes. Dry maculae after the loading treatment, defined as an absence of intra- or sub-retinal fluid upon SD-OCT examination, was attained in 191 eyes (75.8%) in the discovery group and 149 eyes (73.4%) in the replication group (*P* = 0.59). The number of additional treatments during the PRN follow-up period after the loading treatment was 1.98 ± 1.90 in the discovery group and 2.25 ± 2.28 in the replication group (*P* = 0.27).

When the genetic association with dry macula achievement after the loading treatment was evaluated for the discovery stage, no SNP showed a significant association with a genome-wide level P-value (Figure S1A). Since 1 SNP (rs35028047) on chromosome 18 showed a P-value of < 5 × 10^−6^ with an MAF > 0.1 (Table S1), rs35028047 was evaluated in the replication analysis. However, rs35028047 did not significantly associate with dry maculae (*P* = 0.21) in the 205 replication samples, and the odds ratio suggested an opposite effect compared to the results obtained in the discovery stage (Table S2). In addition to rs35028047, 9 SNPs in 8 genetic regions, previously reported to be associated with AMD, were evaluated in association with dry maculae in the discovery stage. None of these SNPs were significantly associated with dry maculae (Table S3).

Because data from previous studies suggested that one-third to one-fourth of patients with AMD do not require additional treatment for one year after 3 monthly loading treatments with ranibizumab^7, 8^, genetic associations with the necessity for additional treatment within one year were investigated. For this analysis, eyes were divided into 2 groups: eyes that had received additional treatment and those without additional treatment during the PRN period. In the discovery stage, no SNPs showed genome-wide level significant associations with the requirement for additional treatment (Figure S1B). Since 1 SNP at nucleotide position 96,157,509 in chromosome 6 showed a P-value of < 5 × 10^−6^ and an MAF > 0.1 (Table S1), this SNP was evaluated in the replication samples. However, our designed probe for this SNP did not work in the TaqMan method. Thus, rs6924709 in the same LD block was chosen for analysis. In the discovery stage, rs6924709 showed a P-value of 4.90 × 10^−6^, but rs6924709 did not show a significant association with the need for additional treatment (*P* = 0.066) in the replication analysis, and the odds ratio suggested an opposite effect occurred between the discovery and replication stages (Table S4). When analyzing the 9 SNPs within the 8 AMD-susceptibility genes, rs10490924 in *ARMS2/HTRA1* showed a significant association with the additional treatment requirement in the discovery stage (*P* = 0.0023, Table 1). Although replication analysis did not show a statistically significant *P*-value for the association between rs10490924 and additional treatment (*P* = 0.14), the odds ratio showed the same direction as did the discovery stage, suggesting that the *P*-value did not reach statistical significance owing to the small sample size, and pooled analysis of the discovery- and replication-stage data showed a strong association between rs10490924 and additional treatment necessity (P = 0.0013, Table 2). The odds ratio suggested that having one G allele lowered the necessity for additional treatment to the 63% level.

**Table 1:** Associations of 9 single-nucleotide polymorphisms in 8 age-related macular degeneration susceptibility genes with an additional treatment requirement after 3 monthly injections of ranibizumab in the discovery stage.

Gene	SNP	1/2	Additional treatment (−) (11/12/22)	Additional treatment ( + ) (11/12/22)	OR (95% CI)	P	OR (95% CI)*	P*
*ARMS2/HTRA1*	rs10490924	G/T	27/25/21	26/72/69	0.501 (0.201–0.338)	0.00053	0.549 (0.323–0.807)	0.0023
*CFH*	rs800292	A/G	10/27/36	12/79/76	0.939 (0.619–1.426)	0.77	0.989 (0.641–1.526)	0.96
	rs1410996	A/G	10/33/30	16/82/69	0.909 (0.606–1.365)	0.65	0.980 (0.634–1.515)	0.93
*CETP*	rs3764261	A/C	1/27/45	8/47/112	0.938 (0.574–1.532)	0.80	0.859 (0.517–1.429)	0.56
*C2/CFB*	rs547154	T/G	0/9/64	0/13/154	0.617 (0.258–1.476)	0.27	0.713 (0.283–1.797)	0.47
*CFI*	rs4698775	G/T	5/28/40	9/60/98	0.866 (0.553–1.356)	0.53	0.919 (0.579–1.457)	0.72
*TGFBR1*	rs334353	G/T	14/34/25	38/77/52	1.145 (0.774–1.696)	0.50	1.216 (0.817–1.809)	0.33
*APOE*	rs4420638	G/A	0/10/60	0/32/130	1.441 (0.689–3.015)	0.33	1.381 (0.626–3.044)	0.41
*VEGFA*	rs943080	C/T	9/35/29	14/76/77	0.793 (0.527–1.195)	0.27	0.791 (0.533–1.292)	0.29

*Adjusted for age and sex, SNP: single-nucleotide polymorphism, OR: odds ratio, CI: confidence interval.

**Table 2:** Replication and pooled analysis of the association between *ARMS2/HTRA1* rs10490924 and additional treatment requirement after 3 monthly injections of ranibizumab.

Stage	1/2	Additional treatment (−) (11/12/22)	Additional treatment ( + ) (11/12/22)	OR (95% CI)	P	OR (95% CI)*	P*
Discovery stage	G/T	27/25/21	26/72/69	0.50 (0.20–0.34)	0.00053	0.55 (0.32–0.81)	0.0023
Replication stage	G/T	15/27/15	22/57/50	0.64 (0.41–1.00)	0.051	0.72 (0.46–1.12)	0.14
Pooled analysis	G/T	42/52/36	48/129/119	0.57 (0.42–0.76)	0.00014	0.63 (0.47–0.83)	0.0013

*Adjusted for age and sex, OR: odds ratio, CI: confidence interval.

We also evaluated genetic associations with visual acuity changes during the 12-month period. In the discovery stage, no SNP showed a genome-wide level significant association with visual acuity changes (Figure S1C), but 4 SNPs showed a *P*-value > 5 × 10^−6^ and an MAF > 0.1, including SNPs at nucleotide positions 3,120,081 on chromosome 1, 171,318,452 on chromosome 3 (rs202164786), 42,770,836 on chromosome 20, and 51,172,460 on chromosome 22 (Table S1). In the replication analysis, no SNPs except for rs202164786 showed a significant association (P > 0.19, Table S5), although rs202164786 could not be examined by our designed probe. Thus, rs9881788 in the same LD block in the *PLD1* gene was evaluated for an association with visual acuity changes. In both the discovery and replication stages, however, rs9881788 did not show a significant association (P > 0.63, Table S6). During the analysis of the 9 SNPs within the 8 AMD-susceptibility genes, rs943080 in *VEGFA* showed a significant association with visual acuity changes (*P* = 0.031, Table 3). However, rs943080 showed an opposite effect with respect to visual acuity (*P* = 0.0065) in the replication stage, and pooled analysis revealed a non-significant association (*P* = 0.86, Table 4).

**Table 3:** Associations of 9 single nucleotide polymorphisms in 8 age-related macular degeneration susceptibility genes with visual acuity change during 12 months of ranibizumab treatment in the discovery stage.

Gene	SNP	BETA (95% CI)	P	BETA (95% CI)*	P*
*ARMS2/HTRA1*	rs10490924	−0.012 (−0.057–0.034)	0.62	−0.013 (−0.058–0.033)	0.58
*CFH*	rs800292	−0.007 (−0.061–0.047)	0.80	−0.009 (−0.063–0.044)	0.74
	rs1410996	−0.011 (0.064–0.041)	0.68	−0.017 (−0.069–0.036)	0.54
*CETP*	rs3764261	−0.004 (−0.066–0.058)	0.89	0.004 (−0.058–0.065)	0.90
*C2/CFB*	rs547154	−0.080 (−0.198–0.039)	0.19	−0.094 (−0.213–0.025)	0.12
*CFI*	rs4698775	−0.033 (−0.090–0.024)	0.25	−0.039 (−0.095–0.018)	0.18
*TGFBR1*	rs334353	0.029 (−0.018–0.076)	0.23	0.024 (−0.023–0.071)	0.33
*APOE*	rs4420638	−0.050 (−0.141–0.040)	0.25	−0.045 (−0.135–0.044)	0.25
*VEGFA*	rs943080	0.062 (0.009–0.114)	0.022	0.058 (0.006–0.110)	0.031

*Adjusted for age and sex, SNP: single nucleotide polymorphism, OR: odds ratio, CI: confidence interval.

**Table 4:** Replication and pooled analysis of the association of *VEGFA* rs943080 with visual acuity changes during 12 months of ranibizumab treatment.

Stage	BETA (95% CI)	P	BETA (95% CI)*	P*
Discovery stage	0.06 (0.01–0.11)	0.022	0.06 (0.01–0.11)	0.031
Replication stage	−0.06 (−0.11– −0.001)	0.047	−0.08 (−0.13– −0.02)	0.0065
Pooled analysis	0.01 (−0.03–0.05)	0.62	0.00 (−0.04–0.03)	0.86

*Adjusted for age and sex, CI: confidence interval.

## Discusson

Genetic associations with AMD treatment outcomes have been investigated in many studies because they can potentially enable personalized AMD therapy. However, most studies were retrospective studies based on candidate genes. In our prospective study performed collaboratively by 13 institutes in Japan, we conducted GWAS on genetic associations with treatment outcomes of anti-VEGF treatment for AMD, but did not find any SNPs with a genome-wide level of significance. To utilize genetic information for personalized medicine, a given SNP should have greatly significant odds ratio because subtle difference are not practical for use making clinical treatment decisions. Furthermore, SNPs with a low MAF are not widely useful considering that the treatment outcome prediction can be applied only patients with the minor allele. In GWASs, a sample size of 250 (as in the present study) should be sufficiently large to discover candidate SNPs with a MAF of 0.2 and an odds ratio of 1.5–2.0. Our negative GWAS results for the treatment outcome suggested that single no SNP has enough power to support personalized medicine for patients with AMD.

Although the *P*-value did not reach the genome-wide significance level, *ARMS/HTRA1* rs10490924 might be useful for predicting whether patients need additional treatment after the initial loading treatment with ranibizumab. When a patient has one G allele in rs10490924, the necessity of additional treatment was predicted to decrease to the 63% level. Because data from previous studies suggested that one-third to one-fourth of patients with AMD do not need additional treatment for one year after loading treatment with ranibizumab^7, 8^, it should be worthwhile to predict the requirement for additional treatment for each patient. However, previous genetic studies did not focus primarily on the additional treatment requirement. Further genetic studies on additional treatment might enable the prediction of which patients will not need additional treatment after loading treatment. Because redundant anti-VEGF treatment might cause RPE atrophy and poor visual prognosis in patients with AMD^6^, such patients should not receive additional treatment after the initial loading injections.

In contrast to the additional treatment requirement, *ARMS2/HTRA1* genotype information was not found to be useful for predicting visual outcome or the achievement of dry maculae after the initial treatment. Previous studies have evaluated associations between SNPs in *ARMS2/HTRA1* and visual outcome after anti-VEGF treatment. Although some reports showed significant associations^25–32^, many studies denied such associations^9, 19, 21–24, 33–39^. In the present study, rs10490924 was not significantly associated with visual acuity changes during the 1-year treatment period when analyzing 255 samples. *ARMS2/HTRA1* lacked sufficient power to predict visual outcomes in personalized medicine for AMD. Previous investigators also examined associations between SNPs in *ARMS2/HTRA1* and the response to ant-VEGF treatment by SD-OCT. The results of most studies did not support their associations^7, 21, 24, 27, 28, 30, 32, 33, 36, 40–42^, while 1 report showed that rs10490924 was significantly associated with the achievement of dry maculae after 3 monthly injections of an anti-VEGF drug^37^. Collectively, previous data and the findings of this study indicate that *ARMS2/HTRA1* is not useful for predicting anatomic outcomes after anti-VEGF treatment for AMD.

Further evaluation of *VEGFA* might be useful for predicting visual outcomes after anti-VEGF treatment for AMD. In the discovery stage of the present study, the T allele in rs943080 was significantly associated with a better visual outcome while it showed significant association to worse visual outcome (odds ratio = 1.08, 95% confidence interval = 1.02–1.39) in the replication stage. Zhao *et al*. also showed that the T allele of rs943080 was significantly associated with poor visual outcome after anti-VEGF treatment for AMD^43^. Although another study denied such an association^33^ and other SNPs in *VEGFA* were shown not to be associated with the response to anti-VEGF treatment^22, 44, 45^, several studies have reported significant associations between *VEGFA SNPs* and the response to anti-VEGF treatment^13, 25, 26, 31, 36, 46, 47^. Because the rs943080 SNP affects the VEGFA-expression level^48^, associations between rs943080 and treatment outcome should be further examined.

Recently, the TAE regimen has been widely used to treat AMD with anti-VEGF drugs. However, some patients should receive redundant injections when treated with the TAE regimen, which can be circumvented by accurately predicting who will not need continuous treatment after the loading injections. Although no single SNP may have sufficient power to predict treatment outcomes, analysis of several SNPs in combination could be useful for personalized AMD medicine.

The limitations of this study are its small sample size and its short-term follow-up period. A GWAS with a larger sample size might find SNPs with lower MAFs and subtle ORs associated with the outcome of anti-VEGF treatment for neovascular AMD. Although no single SNP with a low MAF and subtle odds ratio was identified that could be useful for personalized-medicine, the combination of such SNPs could potentially be useful. Recent studies have shown that the long-term visual outcome of anti-VEGF treatment is not favorable in patients with AMD^1, 49^. Predicting which patients can maintain improved visual function after anti-VEGF treatment would reveal who should be intensely monitored for additional treatment after the initial loading treatment.

In conclusion, we performed a multi-center prospective study to identify genes associated with the treatment outcome after anti-VEGF treatment for AMD. Although no SNP showed a genome-wide level of significance, *ARMS2/HTRA1* might be useful for predicting the requirement for additional treatment after 3 monthly injections of ranibizumab. Further investigation would help increase the accuracy of predicting treatment outcomes in personalized medicine for AMD.

## Methods

### Patients enrolled in the Study

The eligibility criteria included being 50 years of age or older and the presence of treatment-naïve neovascular AMD (1 eye per patient) as evidenced leakage in fluorescein angiography (FA) and fluid or hemorrhaging within or below the macula during spectral-domain optical coherence tomography (SD-OCT) examination, with a best-corrected Snellen visual acuity (VA) equivalent of 20/400 to 20/40 at baseline. Exclusion criteria included any previous treatment for choroidal neovascularization (CNV), vitrectomy, and intraocular lens suture fixation, as well as the presence of angioid streaks, myopic CNV, and vitelliform macular dystrophy. Institutional Review Board/Ethics Committee approval of any relevant details in this study was obtained at each clinical center; Kyoto University Graduate School and Faculty of Medicine, Ethics Committee, Saitama Medical University Hospital Ethics Committee, Ethics Committee of Kobe University Graduate School of Medicine, Fukushima Medical University School of Medicine, Ethics Committee, The University of Tokyo Graduate School of Medicine and Faculty of Medicine, Ethics Committee, Ethics Committee of Seirei Hamamatsu General Hospital, University of Yamanashi Faculty of Medicine Ethics Committee, Gunma University School of Medicine Ethics Committee, Kansai Medical University Medical Ethics Committee, Ohtsuka Eye Hospital Ethics Committee, Miyata Ophthalmic Hospital Ethics Committee, Osaka University Research Ethics Committee, Shimane University Faculty of Medicine Medical Ethics commitee. All study conduct adhered to the tenets of the Declaration of Helsinki. Each patient gave written informed consent for participation in the study.

Before loading treatment, each patient underwent a comprehensive ophthalmologic examination, including measurement of best-corrected VA and intraocular pressure, indirect ophthalmoscopy, slit-lamp biomicroscopy with a contact lens, color fundus photography, SD-OCT examination, fundus autofluorescence, and fluorescein and indocyanine green angiography. Best-corrected VA was measured using a Landolt chart and converted to a logarithm of the minimal angle of resolution (LogMAR) for statistical analysis.

### Treatment

All patients underwent 3 courses of monthly injections of 0.5 mg ranibizumab (Lucentis; Novartis, Bülach, Switzerland) and were monitored monthly afterwards for one year. At every scheduled visit, the best-corrected VA was measured, and slit-lamp biomicroscopy and SD-OCT examination was performed. Re-treatment of each patient was performed if any of the following changes were observed by the evaluating physician during the first year of the study: 1) VA loss of at least 0.1 LogMAR VA with SD-OCT evidence of fluid in the macula, 2) an increase in SD-OCT central retinal thickness of at least 100 µm more than the least thickness observed during the study, 3) new macular hemorrhaging, 4) a new area of classic CNV observed when the evaluating physician performed FA based on suspicion of new classic CNV, or 5) evidence of persistent fluid upon SD-OCT examination 1 month after the previous injection.

### Genotyping

Genotyping was performed with all patients. Genomic DNA was prepared from leukocytes of peripheral blood with a QuickGene-610L DNA extraction kit (Fujifilm, Tokyo, Japan). During the discovery stage, genome-wide genotyping was performed using the HumanOmni2.5–8 BeadChip Kit (Illumina Inc., San Diego, CA). To ensure high-quality genotype data, a series of quality control filters were applied to the raw data, including a MAF cutoff of >0.01 and genotyping success rate of >95%. Genome-wide imputation was performed using the Michigan Imputation Server (https://imputationserver.sph.umich.edu/index.html) with ASN population haplotypes from the 1000 Genomes project (phase 3) as reference sequences. Series of quality-control filters were applied again to the post-imputed dataset, including a MAF cutoff of >0.01, a genotyping success rate of >90%, and an individual call rate of >90%. Quality control was performed using PLINK (version 1.07; http://pngu.mgh.harvard.edu/~purcell/plink; accessed April 11, 2015). During the replication stage, samples were genotyped using TaqMan SNP assays with the ABI PRISM 7700 system (Applied Biosystems Inc., Foster City, CA). We did not consider deviations in genotype distributions from the Hardy–Weinberg equilibrium because all samples were from patients with AMD.

### Statistical Analysis

During the discovery stage, associations of the genotypic distribution of each SNP with the response to the loading treatment (assessed by SD-OCT or the requirement of additional treatment after the loading treatment) were examined by logistic regression analysis, assuming an additive effect of the per minor allele with adjustment for age and sex. Associations of the genotypic distribution of each SNP with the VA change after 12 months were examined using quantitative trait-locus analysis with a linear regression model, assuming an additive effect of the per minor allele with adjustment for age and sex. During the discovery stage, genome-wide significance was defined as P < 5 × 10^−8^. Single nucleotide polymorphism with P values < 5 × 10^−6^ and an MAF of >0.1 were selected as candidates for the replication stage, and differences were considered statistically significant at P < 0.05 thereafter. SNAP software (http://www.broadinstitute.org/mpg/snap/ldsearch.php; accessed April 11, 2015) was used to infer linkage disequilibrium (LD) at the targeted regions. The coordinates presented were from the National Center for Biotechnology Information Build 37 (http://www.ncbi.nlm.nih.gov; accessed April 11, 2015; available in the public domain from the National Center for Biotechnology Information, Bethesda, MD).

The rates of dry macular achievement were compared with Fisher’s exact test, and the numbers of ranibizumab injections were compared with an unpaired-t test between the discovery and replication samples. Differences were considered statistically significant at P < 0.05. The datasets generated during and/or analysed during the current study are available from the corresponding author on reasonable request.

## Electronic supplementary material

Supplementary Information
