# Supplementary material for: A prospective multicenter study on genome wide associations to ranibizumab treatment outcome for age-related macular degeneration
Source: Sci Rep. 2017 Aug 23;7:9196. doi: 10.1038/s41598-017-09632-0 (PMC5569099; doi:10.1038/s41598-017-09632-0)

**A prospective multicenter study on genome wide associations to ranibizumab treatment outcome for age-related macular degeneration**

Kenji Yamashiro, Keisuke Mori, Shigeru Honda, Mariko Kano, Yasuo Yanagi, Akira Obana, Yoichi Sakurada, Taku Sato, Yoshimi Nagai, Taiichi Hikichi, Yasushi Kataoka, Chikako Hara, Yasurou Koyama, Hideki Koizumi, Munemitsu Yoshikawa, Masahiro Miyake, Isao Nakata, Takashi Tsuchihashi, Kuniko Horie-Inoue, Wataru Matsumiya, Masashi Ogasawara, Ryo Obata, Seigo Yoneyama, Hidetaka Matsumoto, Masayuki Ohnaka, Hirokuni Kitamei, Kaori Sayanagi, Sotaro Ooto, Hiroshi Tamura, Akio Oishi, Sho Kabasawa, Kazuhiro Ueyama, Akiko Miki, Naoshi Kondo, Hiroaki Bessho, Masaaki Saito, Hidenori Takahashi, Xue Tan, Keiko Azuma, Wataru Kikushima, Ryo Mukai, Akihiro Ohira, Fumi Gomi, Kazunori Miyata, Kanji Takahashi, Shoji Kishi, Hiroyuki Iijima, Tetsuju Sekiryu, Tomohiro Iida, Takuya Awata, Satoshi Inoue, Ryo Yamada, Fumihiko Matsuda, Akitaka Tsujikawa, Akira Negi, Shin Yoneya, Takeshi Iwata, Nagahisa Yoshimura

**Table S1.** Single-nucleotide polymorphisms with P-values < 5 × 10^-6^ with a minor allele frequency of >0.1 in the discovery stage.

Analysis of associations with dry macula achievement after loading treatment

| CHR | BP | rs | MAF | OR | P |
| --- | --- | --- | --- | --- | --- |
| 18 | 38905735 | rs35028047 | 0.14 | 0.2539 | 4.91E-06 |

Analysis of associations with additional treatment requirement after loading treatment

| CHR | BP | rs | MAF | OR | P |
| --- | --- | --- | --- | --- | --- |
| 6 | 96157509 | NA | 0.12 | 0.2095 | 2.95E-06 |

Analysis of associations with visual acuity changes after 12 months of treatment

| CHR | BP | rs | MAF | BETA | P |
| --- | --- | --- | --- | --- | --- |
| 1 | 3120081 | NA | 0.11 | 0.1706 | 3.11E-06 |
| 3 | 171318452 | rs202164786 | 0.20 | -0.1619 | 2.60E-06 |
| 20 | 42770836 | NA | 0.23 | -0.132 | 3.88E-06 |
| 22 | 51172460 | NA | 0.12 | 0.1659 | 4.71E-06 |

CHR: chromosome, BP: base pair, MAF: minor allele frequency

**Table S2.** Replication analysis of the association with the rs35028047 SNP with the dry maculae achievement after 3 monthly injections of ranibizumab.

|  | TT | TC | CC | OR  (95% CI) | P | OR  (95% CI)* | P* |
| --- | --- | --- | --- | --- | --- | --- | --- |
| dry | 0 | 36 | 112 | 1.52  (0.71–3.28) | 0.28 | 1.69  (0.74–3.83) | 0.21 |
| wet | 0 | 9 | 45 |

*adjusted for age and sex, CI: confidence interval

**Table S3.** Associations of 9 single nucleotide polymorphisms in 8 age-related macular degeneration susceptibility genes with dry maculae achievement after 3 monthly injections of ranibizumab in the discovery stage.

| Gene | SNP | 1/2 | Dry  (11/12/22) | Wet  (11/12/22) | OR (95% CI) | P | OR (95% CI)* | P* |
| --- | --- | --- | --- | --- | --- | --- | --- | --- |
| *ARMS2/HTRA1* | rs10490924 | G/T | 45/76/72 | 11/28/24 | 1.147  (0.761–1.728) | 0.51 | 1.134  (0.773–1.664) | 0.52 |
| *CFH* | rs800292 | A/G | 17/90/86 | 7/25/31 | 1.056  (0.684–1.629) | 0.81 | 1.060  (0.679–1.655) | 0.80 |
|  | rs1410996 | A/G | 22/98/73 | 6/26/31 | 1.348  (0.874–2.078) | 0.18 | 1.380  (0.878–2.169) | 0.16 |
| *CETP* | rs3764261 | A/C | 8/68/117 | 3/14/46 | 1.474  (0.863–2.518) | 0.15 | 1.509  (0.879–2.590) | 0.14 |
| *C2/CFB* | rs547154 | T/G | 0/17/176 | 0/7/56 | 0.783  (0.317–1.934) | 0.60 | 0.763  (0.298–1.953) | 0.57 |
| *CFI* | rs4698775 | G/T | 14/64/115 | 1/28/34 | 1.001  (0.625–1.606) | 1.00 | 0.996  (0.621–1.598) | 0.99 |
| *TGFBR1* | rs334353 | G/T | 41/90/62 | 14/28/21 | 1.005  (0.670–1.506) | 0.98 | 0.993  (0.670–1.473) | 0.97 |
| *APOE* | rs4420638 | G/A | 0/32/151 | 0/10/50 | 1.157  (0.556–2.409) | 0.70 | 1.162  (0.547–2.470) | 0.70 |
| *VEGFA* | rs943080 | C/T | 17/85/91 | 7/34/22 | 0.724  (0.476–1.102) | 0.13 | 0.701  (0.452–1.087) | 0.11 |

*adjusted for age and sex, SNP: single-nucleotide polymorphism, OR: odds ratio, CI: confidence interval

**Table S4.** Analysis of the association of the rs6924709 SNP with additional treatment requirement after 3 monthly injections of ranibizumab.

| Stage | 1/2 | Additional treatment (-)  (11/12/22) | Additional treatment (+)  (11/12/22) | OR  (95% CI) | P | OR  (95% CI)* | P* |
| --- | --- | --- | --- | --- | --- | --- | --- |
| Discovery stage | G/T | 1/30/43 | 0/23/139 | 0.28  (0.16–0.49) | 5.1E-06 | 0.21  (0.11–0.41) | 4.9E-06 |
| Replication stage | G/T | 0/8/49 | 2/30/96 | 2.03  (0.91–4.54) | 0.079 | 2.25  (0.95–5.34) | 0.066 |
| Pooled analysis | G/T | 1/38/92 | 2/53/235 | 0.60  (0.39–0.93) | 0.022 | 0.57  (0.36–0.91) | 0.019 |

*adjusted for age and sex, OR: odds ratio, CI: confidence interval

**Table S5.** Replication analysis of the association of 3 single nucleotide polymorphisms with visual acuity changes during 12 months of ranibizumab treatment.

| SNP | BETA (95% CI) | P | BETA (95% CI)* | P* |
| --- | --- | --- | --- | --- |
| 1:3120081 | 0.008 (-0.07–0.08) | 0.84 | 0.003 (-0.07–0.08) | 0.94 |
| 20:42770836 | 0.02 (-0.05–0.08) | 0.63 | 0.03 (-0.04–0.09) | 0.44 |
| 22:51172460 | 0.06 (-0.02–0.14) | 0.15 | 0.05 (-0.03–0.13) | 0.19 |

*adjusted for age and sex, SNP: single nucleotide polymorphism, CI: confidence interval

**Table S6.** Analysis of the association between the *PLD1* rs9881788 SNP and visual acuity changes during 12 months of ranibizumab treatment.

| Stage | 1/2 | BETA  (95% CI) | P | BETA  (95% CI)* | P* |
| --- | --- | --- | --- | --- | --- |
| Discovery stage | A/C | -0.04 (-0.10–0.01) | 0.09 | -0.04 (-0.09– -0.01) | 0.098 |
| Replication stage | A/C | -0.02 (-0.07–0.04) | 0.55 | -0.01 (-0.07–0.04) | 0.63 |
| Pooled analysis | A/C | -0.03 (-0.07–0.01) | 0.09 | -0.03 (-0.07–0.01) | 0.12 |

*adjusted for age and sex, CI: confidence interval

**Figure S1A.** Manhattan plot for the association with dry macula achievement after three monthly injections of ranibizumab. The horizontal line shows the cutoff value of P = 5 x 10^-8^. The horizontal line shows the cutoff value of P = 5 x 10^-8^.


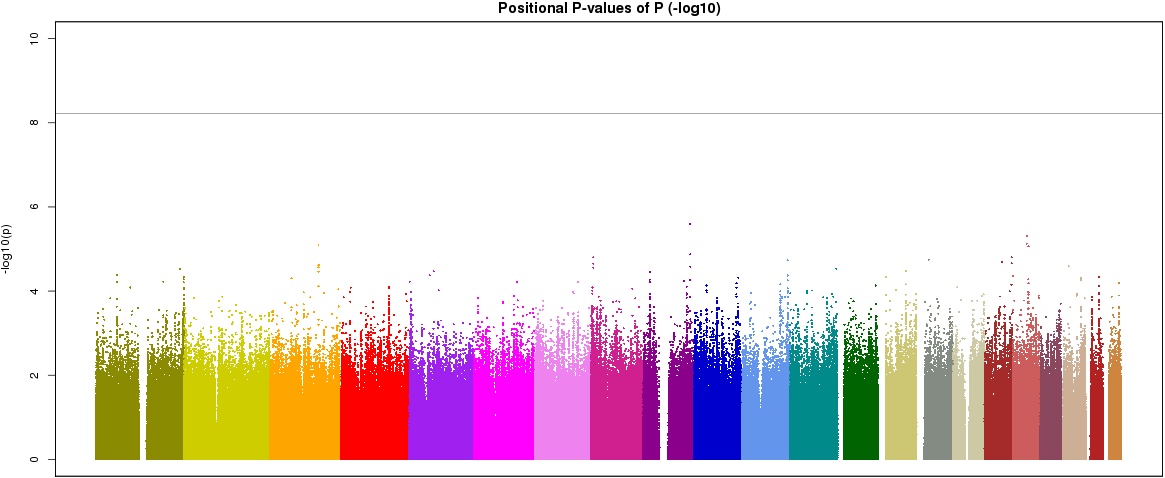


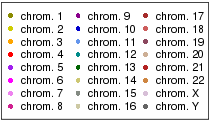


**Figure S1B.** Manhattan plot for the association with additional treatment requirement after the initial treatment. The horizontal line shows the cutoff value of P = 5 x 10^-8^. The horizontal line shows the cutoff value of P = 5 x 10^-8^.


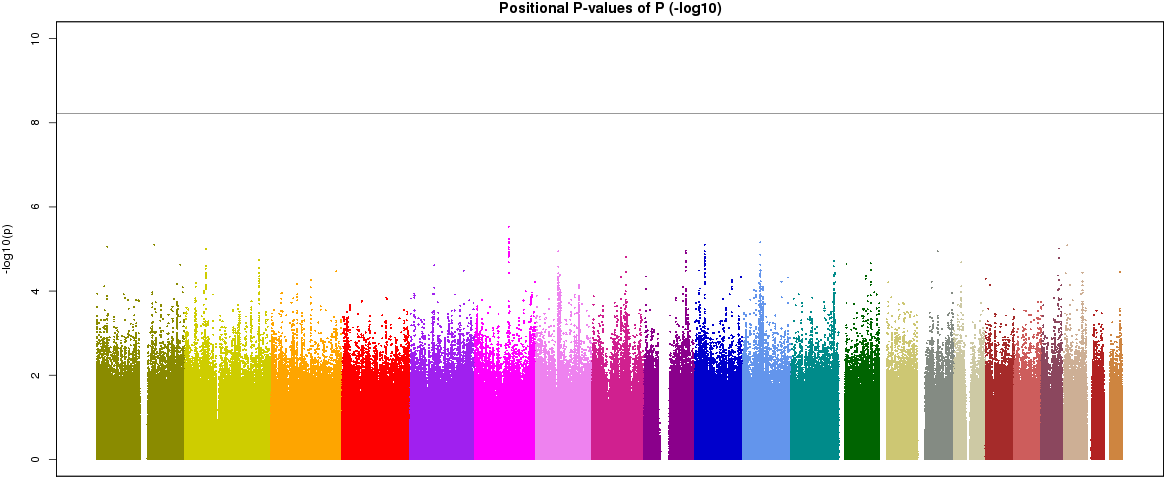


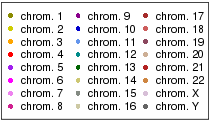


**Figure S1C.** Manhattan plot for the association with visual acuity change at 12th month. The horizontal line shows the cutoff value of P = 5 x 10^-8^. The horizontal line shows the cutoff value of P = 5 x 10^-8^.


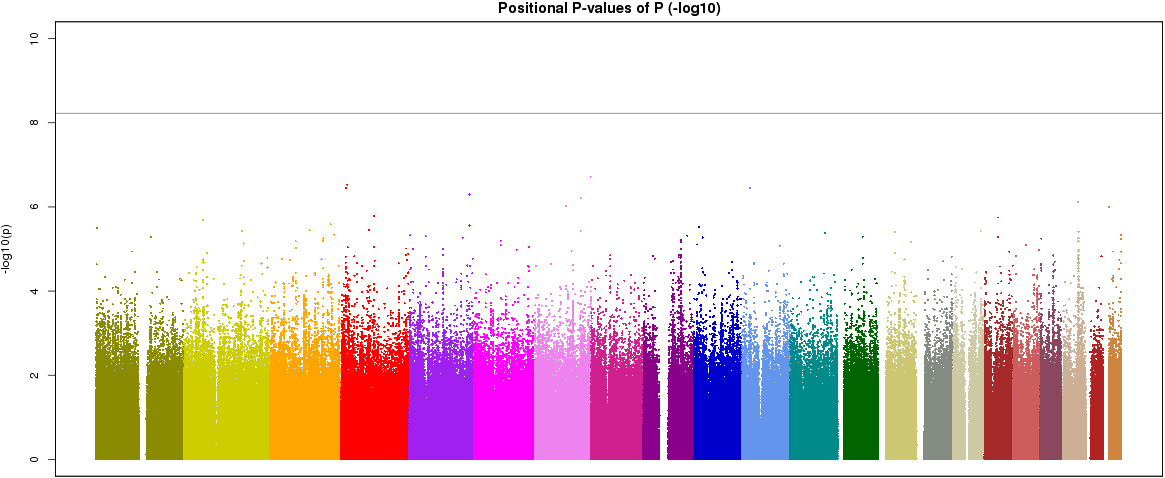


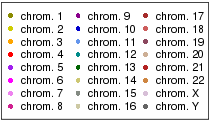

Supplement: Supplementary file 1 — Supplementary Information [file 41598_2017_9632_MOESM1_ESM.doc]
